# Evaluation of aerosols in a simulated orthodontic debanding procedure

**DOI:** 10.1038/s41598-023-32082-w

**Published:** 2023-03-24

**Authors:** Alessandra Pratt, Nile Eckermann, Shankar Rengasamy Venugopalan, Lina Moreno Uribe, Lauren Barlow, Matthew Nonnenmann

**Affiliations:** 1 Department of Occupational and Environmental Health, University of Iowa, Iowa City, IA USA; 2 Department of Orthodontics, University of Iowa, Iowa City, IA USA; 3 School of Dental Medicine, Tufts University, Boston, MA USA; 4 Center for Access and Delivery Research and Evaluation, Iowa City VA Medical Center, Iowa City, IA USA; 5 Department of Environmental, Agricultural and Occupational Health, University of Nebraska Medical Center, Omaha, NE USA

**Keywords:** Dental diseases, Dentistry, Public health, Occupational health

## Abstract

Dental practitioners may be at risk for exposure to severe acute respiratory syndrome corona virus 2 when performing aerosol generating procedures. Though recent evidence suggests that coronavirus may be transmitted through aerosol generating procedures, it is unknown whether common procedures performed in dental clinics generate aerosol. The aim of this study was to simultaneously quantify airborne concentrations of the bacteriophage MS2 near the oral cavity of a dental mannequin and behind personal protective equipment (i.e., face shield) of the practitioner during a simulated orthodontic debanding procedure. A deband was performed eight times on a dental mannequin. Optical particle counters and SKC Biosamplers were used to measure particle concentration and to collect virus aerosol generated during the procedure, both near the oral cavity and behind the orthodontists face shield. A plaque assay was used to determine the viable virus airborne concentration. When comparing the two measuring locations, near the oral cavity and behind the clinician’s face shield, there was no statistically significant difference of virus concentrations or particle size distribution. This study suggests that debanding under these conditions generates live virus aerosol and a face shield does not provide increased protection from virus aerosol, but does provide some protection against splatter during the procedure.

## Introduction

The COVID-19 pandemic has forced many clinicians in the dental and orthodontic field to evaluate occupational exposure risk and whether their current infection control protocols are adequate for protection during aerosol generating procedures (AGP). An AGP is a medical procedure that increases the risk of transmission of infectious aerosols and examples are bronchoscopy, intubation, extubation, and cardiopulmonary resuscitation. While some research has focused on the production of aerosols during dental procedures, questions remain about the transmission of viruses, including SARS-CoV-2, via aerosols during an orthodontic deband procedure (also known as a debond procedure)^1^. Understanding and characterizing these aerosols and their ability to transmit live virus during an orthodontic deband procedure will provide guidance to our profession and decrease the risk of infection to the clinician, staff, and patients in the clinic setting.

Over the past century, respiratory viruses such as Middle East respiratory syndrome coronavirus (MERS-CoV), severe acute respiratory syndrome coronavirus (SARS-CoV), and influenza A have been responsible for the largest viral outbreaks in history. Each of these viruses have placed tremendous stress on healthcare facilities and healthcare workers as well as economies, business owners, and families across the world, but none have had the same effect as SARS-CoV-2. As of today, the COVID-19 pandemic is responsible for over 250 million infections and 5 million deaths worldwide^2^.

Aerosol transmission has been found to be one of the primary ways that SARS-CoV-2 is spread^3–6^. Respiratory aerosols have a broad size range and can be produced from normal daily activities such as breathing, talking, coughing or sneezing^7,8^. These respiratory aerosols can be inhaled, through the nose or mouth, which can cause infection, and is then referred to as aerosol transmission^9,10^. Aerosol transmission is polydispersed particles that are less than 100 µm^11^. Although the SARS-CoV-2 virus has been reported to remain viable in aerosols for up to 3 h, there is still a gap in understanding aerosol transmission especially so in high risk environments^3,12^.

Aerosols are produced during health care procedures, including dental procedures that use high-speed rotary instruments, which can put dentists and orthodontists at an increased risk^13^. Harrel and Molinari reported that ultrasonic, high-speed handpieces, and air–water syringes scalers were sources of aerosol production in a dental office^14^. Another study examined the particle concentrations produced during drilling at a dental office and found that high levels of particles were produced^15^. Particles of this size are of concern due to their ability to facilitate transmission of airborne viruses such as SARS-CoV-2^16^. Lang et al. also came to the conclusion that dental employees are exposed to high concentrations of small particles that pose as a threat for transmitting respiratory diseases^17^.

After orthodontic treatment with bonded fixed appliances has been completed, removal of excess composite from the enamel surface is necessary. This procedure is performed using high or slow-speed handpieces either with or without water. The debanding procedure has been shown to be aerosol generating^18–22^. Specifically, the use of high-speed handpieces has been shown to significantly increase the airborne particle concentration, but uncertainty remains on how the presence or absence of water during this procedure affects aerosol concentration^23^. While information is limited on the size of particles produced during the debanding process, recent studies have shown that particles < 10 µm are generated. In a study by Day et al. the authors examined the aerosols produced during enamel cleanup using high and slow-speed handpieces, with and without water. The results showed that enamel cleanup using slow-speed handpieces, with and without water, produced particulates with a mean size of 5 µm. High-speed handpieces used with and without water produced higher concentrations of particulates as well as particulates with smaller mean diameters, 3 and 1.25 µm, respectively. All four enamel cleanup methods produced particles with a diameter of less than 0.75 µm^24^. Ireland et al. also examined the diameter of particles produced during enamel cleanup using a slow-speed handpiece. The results of this study were similar and found that particles with a diameter of < 2.5 µm were produced during the orthodontic debonding process^25^.

While the production of aerosols in the orthodontic setting has been well studied, the transmissibility of viruses via aerosols produced during a deband procedure is unclear. Questions remain about the viability of virus aerosolized during dental procedures. The goal of this study is to quantify the airborne concentration of live MS2 viral produced during a simulated orthodontic deband procedure without water.

## Materials and methods

### Virus propagation

Bacteriophage MS2 (ATCC 15597-B1) was propagated by inoculating 50 mL of ATCC broth with a single colony of *Escherichia coli* from an agar plate. The liquid medium was incubated overnight at 37 °C. A fresh culture of *E. coli* in ATCC broth was prepared using the overnight culture and incubated at 37 °C for 6 h. The fresh culture, with the virus (100 μL) was inoculated and then incubated 37 °C for 16–24 h. After the incubation period, to separate the host cell debris and the bacteriophage, the culture was centrifuged at 1000 rpm for 20 min. The solution was filtered, with a 0.22 µm pore filter, and the suspension was stored at − 80 °C.

### Deband study design

Ten simulated orthodontic deband procedures were performed using a dental simulation mannequin (A-dec Mobile Simulator Model 4810, A-Dec, Oregon, USA) in a dental college teaching room (room volume 593.8 m^3^). A soft tissue typodont with artificial teeth and an oral cavity cover were used. Prior to the start of each trial, each tooth was etched for 15 s using 35% phosphoric acid (Ultra-Etch, Ultradent, Utah, USA), the etch was rinsed and the teeth were dried. Assure Plus (Reliance Orthodontic Products, Illinois, USA) was placed on the etched surface using a microbrush and each tooth was light cured for 2 s. Transbond XT Light Cure Adhesive Paste (3M, 712-034, California, USA) was placed on the bracket pad (American Orthodontics, Master Series, Wisconsin, USA) and the bracket was positioned on the midfacial surface of maxillary and mandibular teeth. Excess composite was removed prior to light curing for 15 s. The typodont was placed in the simulation mannequin and the brackets were removed using a deband plier (Invecta ODG-344, Pennsylvania, USA). After bracket removal, a curved tip syringe (Covidien Monoject 412, Pearson Dental, California, USA) was used to distributed 1 mL of MS2 across the facial surface of all typodont teeth. Remaining composite was then removed using a high-speed handpiece (Beyes Maxso E600, Electric Micrometer System, Model Number 1600677-011, Bien Air MX2 Microseries, Toronto, Ontario, Canada) with a tapered carbide bur (Brasseler H284K.31.018 FG, Brasseler Dental Instrumentation, California, USA) without water. A polishing bur (Renew Finishing System Point #383, Reliance Orthodontics, Illinois, USA) was used to smooth and polish the facial surface of the typodont teeth. A high-volume evacuator attached to the simulation mannequin followed the high-speed handpiece as excess composite was removed. MS2 bacteriophage was used for this project as a surrogate virus for SARS-CoV-2^26,27^. Stock concentrations of MS2 were 10^9^ plaque forming units (PFU)/mL and stored in a refrigerator in a liquid medium. The oral cavity was cleaned with an antiseptic rinse between trials to ensure that a known amount of MS2 was aerosolized.

### Aerosolization

**Figure 1:**
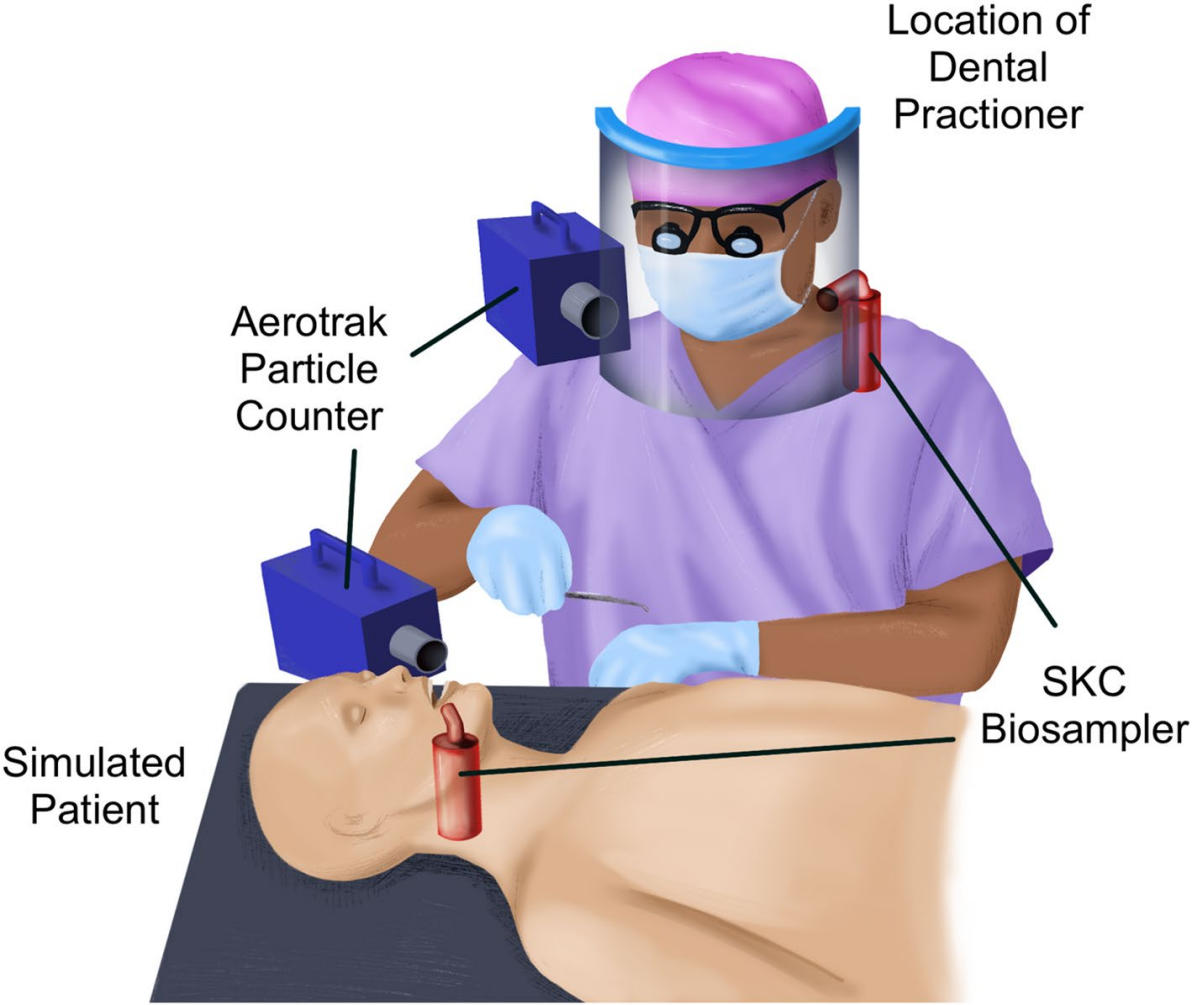
Experimental setup of orthodontic care with active MS2 in the oral cavity of the simulated patient. SKC Biosamplers and optical particle counters simultaneously sampled near the oral cavity of the patient and behind the face shield of the provider.

Eight trials were conducted and, in each trial, aerosolized virus that was generated from inside the oral cavity was analyzed. Each trial consisted of near the mouth and behind the face shield Biosampler measurements. Two SKC BioSamplers (SKC Inc., PA, USA) were used in this experiment to sample for aerosolized particles containing the MS2 bacteriophage. The SKC Biosampler uses inertial impaction to gather particles and incorporate them into its sampling media^28^. Each SKC BioSampler contained 20 mL phosphate-buffered saline (PBS). The BioSamplers were operated at 12.5 L per minute (LPM) (Fig. 1). The BioSamplers were placed next to the particle counters with one placed approximately 30.5 cm (i.e., 12 inches) from the oral cavity and one placed behind the clinician’s face shield. Two optical particle counters (OPC, AeroTrak handheld particle count 9306-V2; TSI Inc., USA) were used to monitor number concentration of aerosols generated during sampling. The particle counters evaluated the concentration at channel sizes of 0.3, 0.5, 1, 3, 5, and 10 μm at 1 min intervals for the duration of the debanding procedure. One OPC was placed 30.5 cm from the oral cavity of the dental mannequin and the other OPC was placed behind the operator’s face shield (Fig. 1).

### Viral strains and growth media

*Escherichia coli* (ATCC 15597, Virginia, USA) was incubated in a liquid media at 37 °C for 6 h. The growth media of *E. Coli* contained 5 mL Supplement and 45 mL tryptic soy broth (TSB). The medium was autoclaved at 121 °C for 20 min. After the autoclaved medium was cooled to 50 °C, the following supplements were added: 1 g glucose (G32045-500.0), 0.294 CaCl_2_ (C614-500), 10 mg thiamine, and 50 mL DI water.

### Double layer plaque assay

For each of the ten deband trials, PBS samples from each BioSampler location, near the oral cavity and behind the face shield, were collected and measured in 50 mL screw cap tubes (Sarstedt Inc., 62.547.100). The samples were concentrated down to less than 1000 µL using centrifugal filters (MilliporeSigma UFC901024, Amicon Ultra-15) which were centrifuged at 4000 rpm for 3 min. A 1.5 mL conical tube was filled with 140 µL of the sample and stored in a − 20 °C freezer. Serial dilution was performed after placing 900 µL of broth and 100 µL of the sample in a 1.5 mL tube and 100 µL of dilution were stored on ice. The plaque assay plates contained two layers of agar; a bottom layer 1.5% agar and a top layer with 0.5% agar. The top agar was boiled at 45 °C for 10 min to stabilize the temperature. A mixture containing 100 µL of serial dilution sample and 10 µL of *E. Coli* were placed in 3 mL of top agar and poured over plaque assay medium in a petri dish (Fisherbrand, FB0875713). All dilutions were plated in triplicate, including the original virus solution which was used in the experiment. Each plate was left at room temperature to solidify for 15 min and was then sealed with parafilm. The plates were incubated at 37 °C for 16–24 h before counting the PFU^29^. The lab also had “lab blanks” throughout the teaching clinic room (sampling) room, ventilation hood, and incubator to ensure no contamination.

### Virus concentration

To concentrate the virus, 15 mL of virus suspension was added to a centrifugal filter device and centrifuged at 4500 g for 10 min at 4 °C to obtain the supernatant.

### Statistical analysis

The concentration of PFU in each aerosol sample were calculated (Eq. 1) and airborne concentration (PFU/m^3^) determined (Eq. 2). A paired t-test was performed comparing the sample concentration near the oral cavity and behind the face shield. Paired t-tests were also performed to compare the particle concentration, between both sampling locations, at each channel size of the OPC.

Equation (1): Plaque assay concentration

1 $$Plaque\; concentration\; \left(PFU\right)=\frac{Average\; PFUs}{(Dilution\; factor* Volume\; plated)}$$

Equation (2): Sample Concentration

2 $$Sample\; concentration=\frac{Plaque\; concentration\; \left(PFU\right)}{Total\; sample\; volume\; \left({m}^{3}\right)}$$

## Results

Prior to the experiment, two preliminary debanding procedure experiments were performed to observe particle concentrations with OPCs to determine if using water would lead to a higher particle concentration compared to not using water. This preliminary trial demonstrated that higher aerosol particle concentrations were generated near the oral cavity when performing a simulated deband without water. Therefore, subsequent experiments using live virus focused on performing debanding procedures without using water. The average time that the deband procedure took was 8 min 22.5 s.

MS2 concentrations were consistently detected near the mannequin oral cavity and behind the face shield in the breathing zone of the operator. The arithmetic mean for viral concentration was 8.46 × 10^6^ PFU/m^3^ (SD = 1.51 × 10^7^) near the oral cavity and 1.15 × 10^6^ PFU/m^3^ (SD = 7.46 × 10^6^) behind the face shield (Table 1). There was no significant difference (*P* = 0.09) in MS2 concentrations between the oral cavity and behind the face shield of the dental care provider.

**Table 1:** MS2 viral concentration (PFU/m^3^) sampled across all trials. The arithmetic mean and standard deviation for each column are provided at the bottom of the table.

Trial	MS2 sample concentration (PFU/m^3^)	
	Near oral cavity	Behind face shield
1	2.39 × 10^6^	2.02 × 10^6^
2	4.54 × 10^7^	3.66 × 10^6^
3	8.62 × 10^6^	1.06 × 10^6^
4	2.05 × 10^6^	5.91 × 10^4^
5	1.24 × 10^6^	8.18 × 10^5^
6	3.98 × 10^6^	4.04 × 10^5^
7	1.05 × 10^6^	3.55 × 10^5^
8	2.95 × 10^6^	8.25 × 10^6^
Mean	8.46 × 10^6^	1.15 × 10^6^
SD	1.51 × 10^7^	1.17 × 10^6^

**Figure 2:**
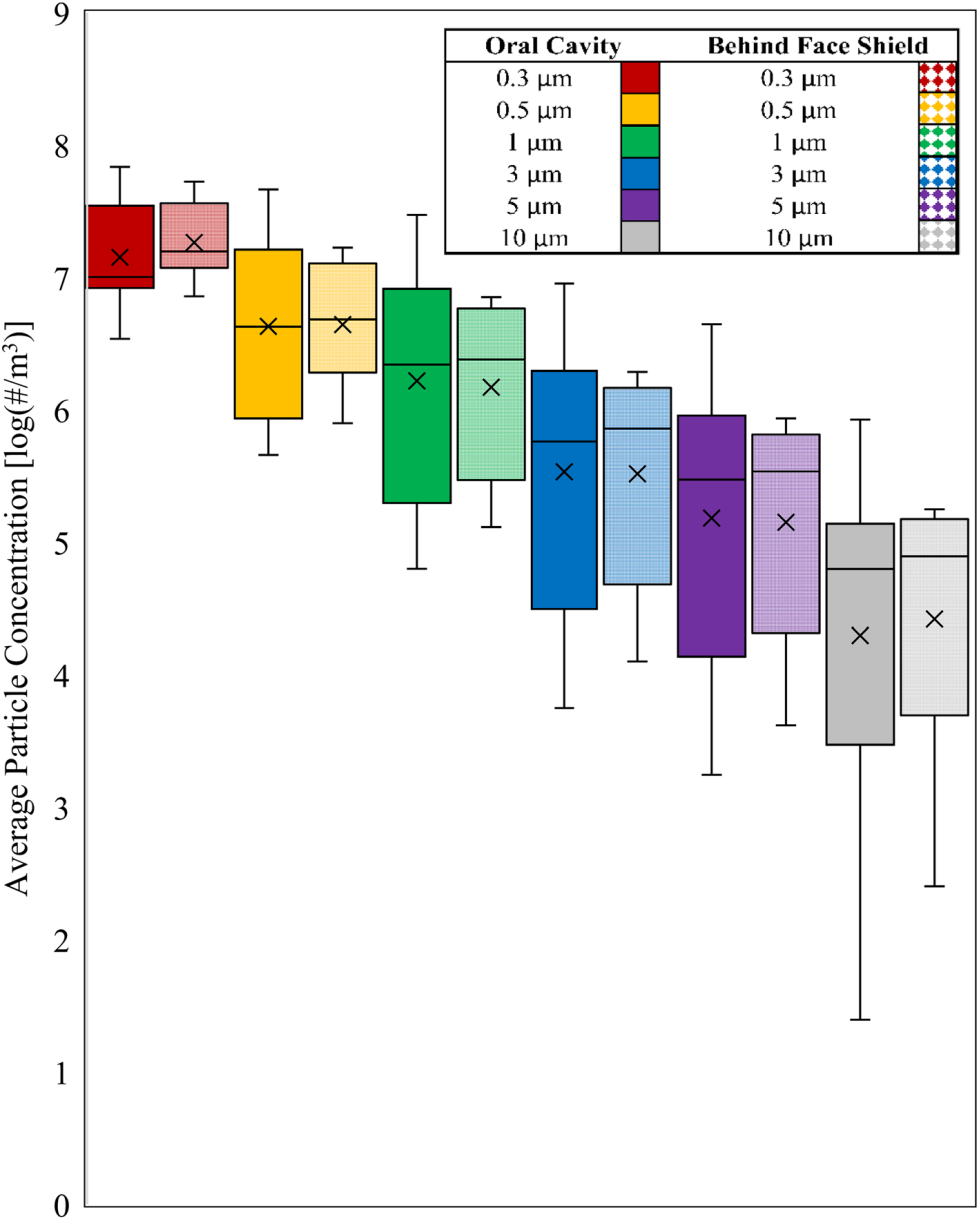
Box plots of arithmetic mean particle concentration (particles/m^3^) at particle sizes ranging between 0.3 and 10 µm across all trials near the oral cavity of the mannequin and behind the face shield of the dental care provider. The error bars represent the standard deviation of concentrations within each particle size bin. No statistically significant difference was observed in particle concentrations behind the face shield compared to near the oral cavity across all particle sizes (*P* values 0.11–0.90).

The arithmetic mean for particle concentration detected by the OPCs was 6.97 × 10^6^ particles/m^3^ near the oral cavity and 5.21 × 10^6^ particles/m^3^ behind the face shield (Table 2). The average particle concentration across all trials at each minute and channel size are represented in Fig. 2. Regardless of sampling location, there was no significant difference (*P* > 0.05) in particle concentrations across particle sizes (0.3–10 µm) (Fig. 2).

**Table 2:** Average particle concentration (particles/m^3^) collected across all trials at each channel size using the optical particle counters near the oral cavity and behind the face shield.

Channel size (µm)	Particle concentration (Particles/m^3^)	
	Near oral cavity	Behind face shield
0.3	2.16 × 10^7^	2.12 × 10^7^
0.5	1.10 × 10^7^	6.26 × 10^6^
1	6.37 × 10^6^	2.69 × 10^6^
3	1.78 × 10^6^	7.10 × 10^5^
5	8.58 × 10^5^	3.17 × 10^5^
10	1.58 × 10^5^	6.87 × 10^4^
Mean	6.97 × 10^6^	5.21 × 10^6^
SD	7.54 × 10^6^	7.46 × 10^6^

## Discussion

The goal of this study was to evaluate the viability of aerosolized particles containing surrogate virus produced during a simulated orthodontic deband procedure without water. We also evaluated the role of a face shield in reducing the concentration of aerosols in the breathing zone of the operator. We observed that a face shield was not effective in reducing the particle concentration or live MS2 virus concentration in the breathing zone of the clinician during debanding procedures. These observations suggest that dental care providers may be at risk for inhalation exposure to viruses during debanding procedures from patients actively shedding virus in oral secretions. This finding is in line with existing literature which has found that dental procedures generate infectious aerosols which may place dental providers at an increased risk for developing a nosocomial infection^30,31^. Furthermore, wearing a face shield provided no significant reduction in viral concentrations in the breathing zone of the dental care provider. The results of this experiment have implications for the health and safety of dental care professionals as the profession addresses risk to providers during the ongoing COVID-19 pandemic and other emerging or endemic viral illness.

Bacteriophage MS2 was used in this study as a surrogate virus for SARS-CoV-2 and has also been used as a surrogate for noroviruses, influenza and coronaviruses^26,32,33^. MS2 is considered was an ideal virus surrogate because it is easily purified, durable, and harmless to humans^27^. MS2 has been used in previous dental procedure research^34^. For our study specifically, detecting aerosolized MS2 demonstrates that the aerosols generated from the debanding procedure are able to reach the breathing zone of the provider. Similar results may be observed for SARS-CoV-2 aerosol, resulting in infection risk.

A bioaerosol sampler is needed in order to capture the aerosolized MS2 produced during the simulated debanding procedure, only particle analysis is not as informative for understanding the risk to providers^35^. The SKC BioSampler is a liquid impingement sampler and was used in this study for bioaerosol collection. Liquid impingers work by directing airflow and impinging particles of a specified size in a liquid media^36^. Multiple studies have used the SKC BioSampler in order to study aerosolized viruses and has also been used to sample aerosolized MS2 in a simulated vomiting study^32,37–39^. However, the SKC BioSamplers collection efficiency of submicrometer and ultrafine particles, which typically contain virus, has been shown to be < 10%^40,41^. However, in a study that examined aerosolized influenza the SKC Biosampler had the highest collection efficiency^28^. Another disadvantage to liquid impinger samplers is that prolonged sampling time can cause evaporation and loss of liquid media, resulting in cell damage which could influence the viability of the virus^36^. This limitation was not of major concern for our study as our sampling times for each trial were less than 10 min. The advantage of using the SKC Biosampler is the flow rate required to operate is similar to ISO standard ventilation rate for performing light work (12.5 LPM and 15 LPM respectively).

When sampling for viruses using a liquid impingement sampler, the use of a liquid sampling media is required. During sampling, the liquid media helps to maintain the viability of the target virus while also allowing immediate sample analysis and virus enumeration. Buffers that have been successfully used in sampling for viruses include Phosphate Buffered Saline (PBS), distilled water, Hanks Balanced Salt Solution (HBSS), peptone broth and transport media nutrient broth^36,39^. PBS has been previously used as impinger sampling media for collected aerosolized MS2^32,40^.

In order to better characterize the clinician’s risk when exposed to aerosolized virus, the use of plaque assays is the most common method in determining virus viability and is also considered to be the gold standard^42^. The infection is allowed to spread to bacterial cells that are immobilized in a layer of agar and these cells eventually undergo cell lysis. Plaques are counted where cell lysis occurs. Each plaque is believed to represent one viral particle from the viral solution, but it is possible cells in that area of the plaque were infected simultaneously by more than one viral particle^29,36^.

While it is well known face shields are effective at protecting against splatter produced during dental procedures, data are limited on what effect face shields have in protecting clinicians against smaller aerosols. One study evaluated the effectiveness of face shields against smaller aerosols, approximately 0–7 µm, during a simulated cough and found that face shields were only effective in blocking 2% of these particles^43^. Another study aimed to measure the concentration of aerosolized particles in the breathing zone of the operator while using various facial protection and suction devices during a simulated dental procedure. Experimental results demonstrated that, with or without the face shield, there was not a significant reduction of aerosolized particles in the breathing zone of the operator^44^. The results of our study were similar and found there was no significant difference in particle concentration or virus concentration near the oral cavity and in the breathing zone of the operator while using a face shield.

As the pandemic continues, these experimental results suggest that using only a face shield will not prevent exposure to virus aerosols so additional protection efforts are needed for infection control strategies. Additional information is needed about exposure concentrations to SARS-CoV-2 resulting in COVID-19 to fully understand the level of risk clinicians and patients experience during dental procedures. The infectious dose for SARS-CoV-2 in humans is currently estimated to have a low inoculum dose of 10 TCID_50_^45^. A previous study evaluating the infectious dose of SARS-CoV found doses corresponding to 10% and 50% illness were 43 PFU and 280 PFU, respectively^46^. These studies examined various inoculation methods including intraocular, aerosol, intrathecal, intragastric, and intranasal. Infection via aerosols was consistently found to have a significantly lower infectious dose and was often associated with more severe disease as smaller aerosols can travel to the lower respiratory tract. The results showed the infectious dose ranged from 2000 PFU in African Green Monkeys to 28,000 PFU in Rhesus Macaques^47^. Another study used sputum data from hospitalized COVID-19 patients and combined this with computational fluid mechanics to estimate an infectious dose between 100 and 300 viral particles for SARS-CoV-2^48^. Our results found that the average PFU/m^3^ near the oral cavity and in the breathing zone of the operator were 8.46 × 10^6^ and 1.15 × 10^6^, respectively. While it is unknown what MS2 airborne concentration would directly correlate to the concentration of aerosolized SARS-CoV-2, our results do demonstrate that debanding has the potential to aerosolize saliva and maintain infectiousness in the breathing zone of the provider.

Our experiment was not without limitations. For example, the room ventilation was not characterized prior to the experiment. However, the experiment was conducted in a clinical teaching room which is likely representative of a clinic environment. The clinic, where orthodontic procedures occur, at the University of Iowa, was not designed to be negative pressure either. Foot traffic was also limited in the teaching clinic to limit the possibility of changing airflow. We compared the difference in mean concentrations that included background particle concentration. We assumed that the background concentration was the same across the face shield. Previous studies that have analyzed aerosolized virus from dental procedures has also successfully been completed in a clinic setting and not a laboratory setting^34,49^.

To limit the potential for contamination across trials, the mouth of the simulated patient was cleaned between trials with an antiseptic rinse. In addition, no more than two trials were performed in 1 day to give time for any aerosolized MS2 to settle between trials. During the analysis, the plaque assay was run in triplicates to reduce the impact of random error. The SKC Biosamplers were autoclaved (sterilized) prior to each use. Plaque assays were also performed on the stock MS2, left in the laboratory, and the experimental MS2, brought and used at the clinic, to ensure that there was no contamination, and that the concentration remained the same.

The experimental setup remained unchanged throughout the study. However, there may be slight differences in the orientation of the provider in the setup across trials. To limit this source of variability, the setup was not moved between trials so that the provider was required to remain in the same location for each trial. However, the provider’s torso, arms, and hand placement many have varied slightly across experiments trials. We believe this error had little effect on the experimental results as the samplers were always oriented to be sampling behind the face shield of the provider. We only sampled at two locations which is a lack of spatial resolution. However, we sampled at the two locations that we believe to cause the greatest risk to the provider; the opening of the mouth where the concentration would be highest as well as the breathing zone of the provider.

To increase generalizability, we conducted the deband with no water, high volume suction, and removing the excess composite with a carbide bur as is done in most orthodontic clinics. There is a small percentage of clinics that use external suction units in addition to the high-volume suction, but this is not standard.

The use of N95 respirators and extraoral suction units have become common practice in dental and orthodontic clinics. While our study and others alike have suggested that face shields do not provide increased protection to aerosolized particles, N95 respirators have been shown to be 95% effective in filtering particles of 0.3 µm^50^. During our simulated deband procedure, our optical particle counters sampled for particles with a size of 0.3 µm and found an average of 2.16 × 10^7^ and 2.12 × 10^7^ particles/m^3^ near the oral cavity and in the breathing zone of the operator were produced, respectively. Remington et al. evaluated the use of extraoral suction units during an aerosol generating procedure and found, when used in conjunction with high speed suction, it was effective in reducing the amount of aerosolized particles that reached the operator^44^. With the development of new variants and the continued spread of SARS-CoV-2 exposure to aerosolized virus is likely, and it is important we remain diligent in our infection control protocols.

## Conclusion

The results of our study suggest using a face shield during a deband procedure does not provide increased protection from live virus aerosols and increased efforts are needed to reduce the clinician’s exposure to virus aerosols and risk of infection. While an infectious dose of SARS-CoV-2 is still undefined, further efforts are needed to guide the dental profession to provide adequate health protection for dental providers and patients from inhalation exposures to virus aerosols.

## Data Availability

The datasets generated during and/or analyzed during the current study are available from the corresponding author on reasonable request.
